# Editorial: Model organisms in embryonic development

**DOI:** 10.3389/fcell.2025.1649186

**Published:** 2025-07-10

**Authors:** Gabriella Lania, Denhi Schnabel, Michael Schubert

**Affiliations:** ^1^ Istituto di Genetica e Biofisica (IGB) Adriano Buzzati-Traverso, Consiglio Nazionale delle Ricerche (CNR), Naples, Italy; ^2^ Instituto de Biotecnología, Universidad Nacional Autónoma de México, Cuernavaca, Mexico; ^3^ Laboratoire de Biologie du Développement de Villefranche-sur-Mer, Institut de la Mer de Villefranche, Centre National de la Recherche Scientifique (CNRS), Sorbonne Université, Villefranche-sur-Mer, France

**Keywords:** embryology, animal model systems, classical model species, alternative model species, evolution of development

“Our real teacher has been and still is the embryo, who is, incidentally, the only teacher who is always right.” — Viktor Hamburger.

Evolution has produced a vast diversity of organisms, and, in order to understand the inner workings of their biology, scientists use model organisms. Findings obtained in model organisms are usually extrapolated and applied to other species, including humans (Nadeau and Auwerx, 2019; Patton et al., 2021; Yamamoto et al., 2024). This practice is possible because the molecular and genetic mechanisms controlling the main biological functions share at least a certain degree of conservation between species (Hall, 2003; Pai and Gilad, 2014; Mosby et al., 2024). Experimental animal model systems have thus been essential not only for uncovering the mechanisms of animal development, metabolism and physiology, but also for advancing agricultural science, biomedical research and the development of treatments for human diseases (Nadeau and Auwerx, 2019; Smith and Rhodes, 2000; Patton et al., 2021; Yamamoto et al., 2024).

Classical animal model systems widely used in developmental studies include the fruit fly *Drosophila melanogaster*, the roundworm *Caenorhabditis elegans* as well as several vertebrates, such as the zebrafish (*Danio rerio*), the clawed frog (*Xenopus laevis*/*Xenopus tropicalis*), the chicken (*Gallus gallus*) and the mouse (*Mus musculus*). While the zebrafish has gained immense popularity in the course of the last few decades, most original discoveries on the biological processes characterizing the earliest stages of development have come from studies using the clawed frog, and groundbreaking experiments in the chicken have revealed how cells and tissues interact during embryogenesis. Regarding the mouse, the fruit fly and the roundworm, they have been instrumental for detailing the spatiotemporal functions of genes during animal development (Barresi and Gilbert, 2023). Despite being incredibly powerful from an experimental point of view, the limited number and restricted phylogenetic coverage of classical animal model systems have proven largely insufficient to describe the diversity of animal developmental mechanisms (Raff, 1992; Haen Whitmer, 2018; Nakamura and Huang, 2025). Recent efforts have therefore focused on the exploration of alternative model organisms, often located at key phylogenetic positions within the animal tree of life, to establish links between the deployment of novel genetic modules during development and the emergence of biological novelties (Stolfi et al., 2010; Steinmetz et al., 2012; Musser et al., 2021).

This Research Topic features examples from both classical and alternative animal models to illustrate the power of established classical models and the usefulness of alternative models for understanding how the evolution of developmental mechanisms drove animal diversification.

Classical model systems featured in articles of this Research Topic include the mouse, which is used by An et al. Their study focuses on blastocyst hatching and its role in implantation of the fetus. Using a high throughput sequencing approach, the authors identify transcriptomic patterns in developing mouse blastocysts that they use to develop a gene expression-based model for predicting implantation success of the blastocyst. Lv et al. use the mouse to assess the role of FGF18 during craniofacial development. In their paper, the authors describe a mouse model allowing the specific increase of FGF18 signaling activity in cranial neural crest cells. The resulting abnormalities in mice are like those of the human Pierre Robin syndrome, a rare congenital birth defect. This work thus demonstrates the need of a tight control of endogenous FGF signaling levels during craniofacial development.

A different classical vertebrate model organism, the clawed frog, is used by Castro Colabianchi et al. to examine the establishment of the embryonic dorsoventral axis. This publication shows that molecules required for ventral patterning of the embryo are asymmetrically distributed in the unfertilized egg, which suggests that the frog egg is prepatterned dorsoventrally prior to fertilization. The establishment of asymmetries is an important feature of body axis formation and found in different animal species and developmental contexts. The work by Guichard et al. provides another example for this, focusing on the emergence of left-right asymmetries during brain development of the lamprey, a jawless vertebrate and an alternative vertebrate model organism. The authors are interested in asymmetric development of the habenula, a bilateral epithalamic structure. Using a transcriptomic approach, they first identify novel markers of different habenular territories and subsequently characterize the development of the lamprey habenula, allowing them to describe a significant asymmetric temporal regulation of habenular territories. Altogether, this article highlights the importance of the lamprey as a model for understanding the evolution of brain asymmetries in vertebrates.

One article of the Research Topic addresses brain development in the fruit fly *D. melanogaster*, a classical invertebrate model organism. More specifically, Barthel et al. study the role of two components of the transcriptional elongation machinery, Spt4 and Spt5, during mushroom body development. The mushroom body is a paired structure in the fruit fly brain required for processing sensory inputs. Taking advantage of the genetic tools available for this model, the authors demonstrate that, in the developing fruit fly mushroom body, Spt4 and Spt5 control both cell proliferation of neural progenitors and remodeling of certain axonal projections. The cephalochordate amphioxus (*Branchiostoma floridae*), an invertebrate chordate model, is used by Kozmik and Kozmikova to study the function of Pax6 during nervous system development. They show that deletion of two amino acids in the conserved paired domain of Pax6 using the CRISPR/Cas9 approach is sufficient for reducing the ability of Pax6 to activate target genes. In these Pax6 mutants, gene expression is altered in the anterior central nervous system, which is consistent with a conserved role for Pax6 in chordate brain regionalization. Mikula Mrstakova and Kozmik use an alternative vertebrate model system for their work, the medaka fish (*Oryzias latipes*), to test the hypothesis of a quasi-universal requirement for Pax6 during animal eye development. Their results reveal significant functional differences between medaka and mouse Pax6 genes, which allows the authors to retrace the evolutionary history of the roles of Pax6 during vertebrate eye development.

The Research Topic further includes studies using alternative animal models covering a wide variety of different animal taxa. Romanova et al., for example, report on long-term cultures of placozoans, early-branching metazoans characterized by an extremely simple body plan. Detailed analyses of the placozoan *Trichoplax adherens* reared for several years in the laboratory reveal surprisingly complex population dynamics and provide evidence that placozoans might have magnetoreception. This work thus reveals novel aspects of placozoan biology and further establishes these animals as alternative model systems in ecology, systems biology and evolution. Wanninger and Schwarze have contributed a review focusing on the usefulness of the quagga mussel (*Dreissena rostriformis*) as an alternative model system in ecology and evolutionary biology. The article discusses the resources currently available for this bivalve mollusk and covers recent advances of our understanding of quagga mussel biology, with special focus on the morphology and molecular biology of development as well as on the molecular and cellular mechanisms underlying physiological adaptations. Anselmi et al. use different invertebrate chordate models, namely tunicates, for a comparative study of developing solitary (*Ciona robusta*) and colonial (*Botryllus schlosseri*) species. While solitary tunicates are oviparous with fertilization and development taking place outside the parent body, colonial tunicates are ovoviviparous with adults retaining the embryos inside their body. The authors thus first describe a method for *in vitro* culturing of *B. schlosseri* embryos and subsequently characterize the development of this colonial tunicate. Finally, they compare the time courses of *C. robusta* and *B. schlosseri* embryology and identify significant heterochronic shifts between the two tunicate species.

Taken together, the articles of this Research Topic nicely illustrate how the combination of classical and alternative model organisms enrich our understanding of animal development. It is thus important to keep pushing the boundaries by both expanding the experimental toolkit and enlarging the species sampling to more comprehensively cover the true diversity of life.
